# Assessment of Clinician Decision-making on Cancer Screening Cessation in Older Adults With Limited Life Expectancy

**DOI:** 10.1001/jamanetworkopen.2020.6772

**Published:** 2020-06-08

**Authors:** Nancy L. Schoenborn, Jacqueline Massare, Reuben Park, Cynthia M. Boyd, Youngjee Choi, Craig E. Pollack

**Affiliations:** 1 Department of Medicine, Johns Hopkins University School of Medicine, Baltimore, Maryland; 2 Department of Biology and Public Health, Johns Hopkins University, Baltimore, Maryland; 3 Department of Health Policy and Management, Johns Hopkins Bloomberg School of Public Health, Johns Hopkins University, Baltimore, Maryland

## Abstract

**Importance:**

Despite clinical practice guidelines recommending against routine cancer screening in older adults with limited life expectancy, older adults are still frequently screened for breast, colorectal, and prostate cancers.

**Objective:**

To examine primary care clinicians’ decision-making on stopping breast, colorectal, or prostate cancer screening in older adults with limited life expectancy.

**Design, Setting, and Participants:**

In qualitative interviews coupled with medical record–stimulated recall, clinicians from 17 academic and community clinics affiliated with a large health system were asked how they came to specific cancer screening decisions in 2 or 3 of their older patients with less than 10-year of estimated life expectancy, including patients with and without recent screening. Patients were surveyed by telephone. Data collection occurred between October 2018 and May 2019.

**Main Outcomes and Measures:**

Clinician interviews were audio-recorded and transcribed verbatim. Transcripts were analyzed with qualitative content analysis to identify major themes. Patient surveys assessed perception of cancer screening decisions, importance of clinician recommendation, and willingness to stop screening.

**Results:**

Twenty-five primary care clinicians (mean [SD] age, 47.1 [9.7] years; 14 female [56%]) discussed 53 patients during medical record–stimulated recall, ranging from 2 to 3 patients per clinician; 46 patients and 1 caregiver (mean [SD] age 74.9 [5.4]; 31 female [66%]) participated in the survey. Clinician interviews revealed 5 major themes: (1) cancer screening decisions were not always conscious, deliberate decisions; (2) electronic medical record alerts were connected with less deliberate decision-making; (3) cancer screening was not binary and clinicians often considered other options to scale back screening without actually stopping; (4) in addition to patient characteristics, clinicians were influenced by patient request and anecdotal experiences; and (5) influences outside of the primary care clinician-patient dyad were important, such as from specialists and patients’ family or friends. Patient surveys asked approximately 64 cancer screening decisions of 47 patients. Patients did not recall approximately half (31 of 64) of their cancer screening decisions. Among those with recent screening, the mean score for willingness to stop screening was 3.2 (95% CI 2.5-3.9) on a 5-point Likert scale (with 1 indicating “extremely unlikely” and 5 indicating “extremely likely”). In most screening decisions that involved specialists (13 of 16), patients valued specialists’ recommendations over those of primary care clinicians.

**Conclusions and Relevance:**

Cancer screening decision-making is complex. Study findings suggest that strategies that facilitate more deliberate decision-making may be important in cancer screening of older adults with limited life expectancy.

## Key Points

**Question:**

How do clinicians and patients think about stopping cancer screening in older adults with limited life expectancy?

**Findings:**

In this mixed methods study with 25 clinicians and 47 patients, cancer screening decisions were found to be not always conscious or deliberate. When the decisions were deliberate, they were associated with not only patient characteristics, but also subjective factors such as patient request, clinicians’ anecdotal experiences, and the patients’ family and friends.

**Meaning:**

In this study, findings suggest that strategies to facilitate more deliberate decision-making may be important in cancer screening of older adults with limited life expectancy.

## Teaser

This qualitative study uses medical record–stimulated recall to examine primary care clinicians’ decision-making on stopping cancer screening in specific older patients with limited life expectancy.

## Introduction

Studies report that the benefits of screening for breast, colorectal, and prostate cancers may be delayed for up to 10 years while risks and burdens of screening occur in the short term.^1,2,3,4,5,6,7,8^ Despite clinical practice guidelines recommending against routine cancer screening in older adults with less than 10 years of life expectancy,^9,10,11,12,13,14,15^ these older adults often still receive screening, with rates as high as 55% in national studies.^16,17,18,19^

Clinician recommendation is a substantial factor in patients’ cancer screening decisions.^19,20,21^ Although studies have shown that clinicians were less likely to recommend screening if patients were older or experiencing poor health, a number of clinicians in these studies still recommended screening despite older age and poor health.^22,23,24,25,26^ Although potential barriers to stopping screening when patients have limited life expectancy have been described,^27,28^ such as difficulty in estimating life expectancy, validated prognostic tools^29^ have not been associated with improvement in screening practices. Better understanding of the range of factors, including facilitators as well as barriers, associated with clinician recommendation of screening cessation, and how clinicians weigh these factors is key to informing interventions to reduce overscreening. Further, previous research often relied on the use of hypothetical scenarios which may not reflect actual decision-making and behavior.^22,23,26,30,31,32^ In addition, as the decision to stop screening involves interaction between clinicians and patients, simultaneous investigation of both perspectives can offer important insights but has not been previously examined.

To address these knowledge gaps, we used the method of medical record–stimulated recall to examine primary care clinicians’ decision-making on stopping cancer screening in specific older patients with limited life expectancy. Medical record–stimulated recall explores the rationales behind clinician recommendations to actual patients and provides more reliable data than responses to hypothetical clinical scenarios.^33^ We also surveyed the patients to obtain complementary perspectives on their cancer screening decision-making.

## Methods

We conducted semistructured in-depth interviews coupled with medical record–stimulated recall, with primary care clinicians in which we asked about cancer screening decision-making in older adults, including the specific cancer screening decisions in 2 or 3 of their older patients. We also surveyed these patients by telephone. This project was approved by a Johns Hopkins School of Medicine institutional review board. We obtained oral informed consent by telephone from patients and written informed consent from clinicians. We provided a $150 gift card per clinician and a $20 gift card per patient.

### Patients and Recruitment

We recruited clinicians via email from 3 academic primary care clinics, 3 geriatric clinical programs, and 20 community primary care clinics affiliated with Johns Hopkins Medicine. We sought clinicians who provided primary care to older adults. We used maximum variation sampling to recruit clinicians who varied in age, sex, clinician type, and specialty, as variations in these characteristics may be associated with different cancer screening practices.^23,34,35^

Once a clinician expressed interest in the study, we reviewed the clinician’s patient records over the previous 6 months to identify patients for medical record–stimulated recall. We included patients who were at least 66 years old, eligible for screening based on history, and had less than 10 years of predicted life expectancy based on an estimation algorithm using age, sex, and comorbidities abstracted from the medical record.^36^ We aimed to discuss 2 to 3 patients per clinician, including at least 1 patient who had breast, colorectal, or prostate cancer screening in the previous year and at least 1 patient without recent screening or had been documented as stopped routine cancer screening.

Prior to the clinician interviews, patients eligible for medical record–stimulated recall were invited to participate in a telephone survey. For patients with cognitive impairment, defined as missing 2 or more points on a validated 6-question cognitive screen,^37^ the patients’ caregivers were invited to participate on the patients’ behalf if the caregivers usually accompanied patients to primary care visits. We excluded those who did not speak English, had severe hearing impairment, could not provide informed consent, or failed cognitive screen and had no eligible caregiver. With clinicians for whom we were able to survey at least 2 eligible patients in advance, these patients were discussed as part of medical record–stimulated recall in the clinician interviews. Otherwise, we also selected patients for medical record–stimulated recall based on medical record review so that we discussed at least 2 patients per clinician.

### Clinician Interview Guide and Patient Survey

The interview guide was piloted with 2 primary care faculty to ensure clarity and appropriateness (eAppendix 1 in the Supplement). The interview questions and the patients to be discussed were sent to the clinician 1 to 2 days in advance. At the interview, we stated that we were interested in how clinicians considered breast, colorectal, and prostate cancer screenings in patients 65 years and older. Regarding each specific patient, we asked how the clinician arrived at the cancer screening decisions in that patient. We then explored in general the clinicians’ cancer screening decision-making in older adults, focusing on the barriers and facilitators to stopping screening. The interviews were semistructured and allowed for new topics to emerge.

The survey (eAppendix 2 in the Supplement) assessed patient-reported trust of the clinician,^38^ whether the patient recalled making a decision about breast, colorectal, or prostate cancer screening with their clinician, satisfaction with the decision, whether a specialist was involved in cancer screening, and the importance of the recommendations from the primary care clinician and from the specialist. Among those without recent screening, we asked if they had decided to stop routine screening. Among those with recent screening, we asked of their willingness to stop screening if recommended by their primary care clinician and whether such a recommendation would affect their trust of the clinician.

### Data Collection and Analysis

One investigator (N.L.S.) with prior qualitative research experience conducted the clinician interviews in person. The investigator, who was a physician in the same health system, was acquainted with some of the participants. Clinician interviews occurred privately in conference rooms or offices and were audio-recorded. Another investigator (J.M.) conducted the patient surveys by telephone. Data collection occurred between October 2018 and May 2019.

Patient survey results were analyzed descriptively. The clinician interview audio recordings were transcribed verbatim and analyzed using textual data analysis software (Atlas.ti, version 8.0; Atlas.ti). The transcripts were continuously reviewed and assessed for the new ideas; data collection continued until no new ideas emerged and theme saturation was reached.^39^ Standard techniques of qualitative content analysis were used to code the transcripts.^39,40,41^ We developed a preliminary coding scheme based on the interview guide and, informed by grounded theory, used open coding to allow inductive identification of new themes using the constant comparative approach in which we iteratively compared information within and across interviews.^39,41^ Each transcript was coded independently by at least 2 of the investigators (N.L.S., J.M., and R.P.). Differences were reconciled by consensus.

## Results

Twenty-five primary care clinicians from 17 clinics participated in the study (Table 1). Most were physicians (19 of 25 [76%]) and women (14 of 25 [56%]), with mean (SD) age of 47.1 (9.7) years. Clinician specialties included 13 internal medicine (52%), 6 family medicine (24%), 2 combined medicine and pediatrics (8%), and 4 geriatric medicine (16%). Mean (SD) timing of interviews was 45 (10) minutes.

**Table 1.** Demographic Characteristics of 25 Primary Care Clinicians

Characteristic	No. (%)
Age, mean (SD), y	47.1 (9.7)
Female sex	14 (56)
Race	
White	15 (60)
African American	5 (20)
Asian	4 (16)
Other	1 (4)
Degree	
MD (doctor of medicine)	16 (64)
DO (doctor of osteopathic medicine)	3 (12)
CRNP (certified registered nurse practitioner)	5 (20)
PA (physician assistant)	1 (4)
Time since completing training, mean (SD), y	16.1 (10.0)
Specialty	
Internal medicine	13 (52)
Family medicine	6 (24)
Combined medicine and pediatrics	2 (8)
Geriatric medicine	4 (16)
Clinic site	
Urban	13 (52)
Suburban	12 (48)
Clinic type	
University-affiliated clinic	8 (32)
Community group practice	14 (56)
House-call program	1 (4)
Program for All-inclusive Care of Elderly (PACE)	2 (8)
No. of single 4-h clinic sessions, mean (SD), per wk	7.0 (2.3)
Proportion of older patients in patient panel	
<25%	6 (24)
25%-49%	10 (40)
50%-74%	3 (12)
>74%	6 (24)

We called 162 patients identified from the medical record and were able to reach 101. Fifteen of 101 were excluded for reasons of hearing impairment, failed cognitive screen, or inability to provide informed consent (eFigure in the Supplement). Of the remaining 86, 46 patients and 1 caregiver (55%) participated in the survey (Table 2), ranging from 1 to 3 patients per clinician. Those who declined to participate were older (mean age 77.6) compared with participants whose mean (SD) age was 74.9 (5.4) years. Most patients were women (31 of 47 [66%]) and white (36 of 47 [77%]). For the 4 clinicians who each had less than 2 participating patients, we selected additional patients for discussion based solely on medical record review. A total of 53 patients were discussed during medical record–stimulated recall. Mean (SD) time between the patients’ most recent clinic visits and the clinician interviews was 44 (32) days.

**Table 2.** Demographic Characteristics of 47 Patients^a^

Characteristic	No. (%)
Age, mean (SD), y	74.9 (5.4)
Age group, y	
65-69	7 (15)
70-74	16 (34)
75-79	17 (36)
80-85	5 (11)
>85	2 (4)
Female sex	31 (66)
Race	
White	36 (77)
African American	10 (21)
Other	1 (2)
Estimated 10-y mortality risk^b^	
>50%-60%	16 (34)
>60%-70%	13 (28)
>70%-80%	7 (15)
>80%-90%	6 (13)
>90%	5 (11)
Educational level	
Did not complete high school	3 (7)
Completed high school	10 (22)
<4 y college	14 (30)
College graduate or postgraduate degree	19 (41)
Duration of relationship with clinician, mean (SD), y	3.6 (3.5)
Trust in clinician, mean (SD)^c^	4.8 (0.5)

^a^ For 1 of the patients, the caregiver participated in the survey on behalf of the patient.

^b^ Using the Tan et al^36^ algorithm, patients are eligible only if estimated 10-year mortality risk was >50%, which is the equivalent of a median life expectancy of <10 years.

^c^ Participants were asked about the extent of their agreement with the statement, “All in all, you have complete trust in your doctor.” A rating of 1 indicates strongly disagree, and a rating of 5 indicates strongly agree.^38^

Content analysis revealed 5 major themes. Themes are presented with representative quotations.

### Theme 1: Lack of Deliberation in Cancer Screening Decisions

Cancer screening decisions were not always conscious or deliberate. For 26 of the 53 patients (49%), their clinician either described the cancer screening decision as not a conscious decision or was not able to recall how the decision was made even after medical record review. This was true in patients with and without recent screening. One clinician said, “Sometimes decisions are made in a hurry in a rushed clinic, and they are not really thought through. It would be foolish for me to pretend that every decision I make on these patients is the product of a rational thought process and shared decision making.”

Sometimes after reviewing patient medical records, clinicians reported that they were not sure how they came to their decisions and, without prompting, said that they should have made the opposite decision in some patients. One clinician commented about a patient without recent colorectal cancer screening, “He should still be screened for colon cancer and I don’t know why it was not on there.” Another clinician said about a patient recently referred for screening, “I referred her for a colonoscopy…. Now I am thinking that the decision was incorrect…. I am not sure how I came to that decision right now.”

### Theme 2: Electronic Medical Record Alerts and Decision-making

Electronic medical record (EMR) alerts were associated with less-deliberate decision-making. Clinicians described a few different contexts in which less deliberate decisions occurred. For those patients who were screened, screening tests were often ordered from routine process or were triggered by EMR alerts, which prompted clinicians about breast and colorectal cancer screenings for patients up to age 75. For patients not recently screened, lack of screening was frequently attributed to the absence of EMR alerts or that cancer screening was simply not mentioned.

Several clinicians commented on how it was easy to order screening in response to EMR alerts without much deliberation, such as “You may just click off [the alert], like: ‘Oh you are due for a mammogram’, and order it without really considering whether or not it’s appropriate.” Other clinicians commented on how reliance on EMR alerts contributed to omissions, such as “What happened in all honesty is I probably would offer him a screening… but it disappeared from the prompt.” One clinician commented that she would trust the EMR alerts over her own judgement, “Even if I thought you were due for this [screening]… if [an EMR alert] doesn’t pop up, then I say: ‘OK it must have been done or must be this patient doesn’t need it’… I trust the power to be to have the system set to not miss anything or not over prompt.”

### Theme 3: Nonbinary Screening Decisions

We found that the cancer screening decision was not limited to the binary options of continuing vs stopping. Clinicians often considered other option to reduce screening without actually stopping. Rather than stopping cancer screening altogether in patients who were older or had significant health concerns, clinicians considered other alternatives such as less frequent screening, relying on physical exam for screening (eg, breast and prostate exams), or using less invasive screening tests (eTable in the Supplement). Another commonly mentioned approach was deferring the decision to a later time, often in patients with more active health issues.

### Theme 4: Patient Request and Anecdotal Experiences as Decision-making Factors

When asked about the factors associated with their decision to stop routine cancer screening in older adults, clinicians mentioned a number of objective factors that informed the benefits and harms of screening. These factors included patients’ age, health status, functional status, quality of life, risks from the screening test, baseline risk of cancer, family history for cancer, and the patients’ ability to tolerate further tests or treatment.

Clinicians also mentioned that other less evidence-based factors, such as patient request and anecdotes, were associated with their decisions. Patient request for testing was a substantial factor. Most clinicians would give in to the patients’ request for screening even if they otherwise would have stopped screening, “It is not necessarily evidence-based medicine, but if people feel like taking good care of their health and having their doctor care about that means they get an annual mammogram, I’m fine with that…. For somebody that I didn’t think [mammography] was indicated I still wouldn’t say no.” In addition, clinicians mentioned various anecdotal experiences involving patients or their personal experiences that were factors in their cancer screening practice (Table 3).

**Table 3.** Anecdotal Experiences Associated With Clinician Cancer Screening Decisions (Theme 4)

Context	Type of anecdote	Example quotation
In support of continued screening	Patient who did well with cancer treatment	“I had a patient that was treated for colon cancer at 100 years old. Seeing how well she did…[screening] may not be a bad idea.”
	Patient who did poorly from advanced cancer	“I had a patient diagnosed with breast cancer in her 80s…the mass was nearly coming out of her breast and…she didn’t do well…I think it’s not a bad idea to go ahead and get [screened].”
	Nearly missed diagnosis	“What would influence me is if someone wanted a test…and then something was positive. I’d say ‘Wow, I was not gonna order that…maybe I should go with what the patient wants.’”
	Cancer in family member	“My aunt at age 82 had [breast cancer]….So that may factor in perhaps, my own family may factor into [my decisions].”
In support of screening cessation	Patient who experienced psychological harm from screening	“She had a tiny lesion, biopsy was ductal carcinoma…[and was told] she must have a lumpectomy or mastectomy. She was very upset, just couldn’t sleep, just so emotionally upset….[Another doctor] told her…repeat a mammogram in a year and see if it’s changed…All of a sudden, she slept OK and she’s feeling better. And it turned out she lived for years and we never did anything else.”
	Patient who regretted cancer treatment	“I can think of a few people who…view it as—boy, I got treated for this cancer which I never would have known that it was really going to hurt me …I did the surgery and I feel miserable as a result.”
	Personal experience with false positives	“I have a husband who has had 3 biopsies. They all came out negative [but] we are worried the entire time.”

### Theme 5: Factors Outside the Clinician-Patient Dyad

Clinicians commented that they had less control over screening decisions when patients saw a specialist, such as “Some patients seem to be on sort of breast cancer [screening] auto pilot…the breast cancer screening folks have taken over that and I tend to have less of a role. The same thing can be with colonoscopy, some people have an outside gastroenterologist who has gotten them set up on a q 5 year screening plan and I’m just sort of along for the ride.”

Clinicians also noted that patients’ family and friends were important in the patients’ decision-making. One clinician said, “I feel like I am less likely to impact a woman’s decision to have a mammogram than her sister who caught a breast cancer on it or her neighbor who is convinced that her mammogram gave her cancer. That stuff is hard to break through.”

### Patient Survey Results

Among the 47 patients who participated in the survey, 64 cancer screening decisions were discussed, as some patients were eligible for more than one screening based on medical history and prior cancer screening (Table 4). These were the same screening decisions discussed during medical record–stimulated recall with their clinicians. Similar to clinicians, patients did not recall approximately half of the screening decisions (31/64).

**Table 4.** Screening Decisions of 47 Patients^a^

Response	No. (%)
Type of screening decision	
Breast cancer	29 (45.3)
Colorectal cancer	21 (32.8)
Prostate cancer	14 (21.9)
Screening status	
Received screening in past year	19 (29.7)
Clinician ordered screening in past year which patient planned to complete	7 (10.9)
No recent screening (or order) but had not decided to stop screening^b^	16 (25.0)
Stopped screening	22 (34.4)
Did not recall making cancer screening decision	31 (48.4)
Satisfaction with screening decision, mean (SD)	4.5 (0.9)
Willingness to stop screening if recommended by clinician, mean (SD)^c^	
Among those	
Screened or ordered screening in past year	3.2 (1.6)
Without recent screening but had not stopped screening	3.8 (1.3)
Decisions for which patients saw relevant specialists^d^	16 (25.0)
Among these, decision in which patients valued specialist’s opinion over primary care clinician’s	13/16 (81.3)

^a^ Of the 47 patients who participated in the telephone survey, we asked about 64 cancer screening decisions in the survey, as some patients were eligible for more than one screening based on medical history and prior cancer screening. The unit of analysis for the responses in this table was each screening decision (n = 64).

^b^ Patients were in this category if they still planned to get screened or were not sure about stopping screening.

^c^ As measured on a 5-point Likert scale, ranging from 1 (extremely unlikely) to 5 (extremely likely) to stop screening.

^d^ Patients were asked if they were seeing gynecologists regarding breast cancer screening, gastroenterologist regarding colorectal cancer screening, or urologist regarding prostate cancer screening.

Among patients who were screened or ordered screening in the past year, willingness to stop screening, if recommended by their primary care clinician, had a mean score of 3.2 (95% CI 2.5-3.9) on a Likert scale, in which 1 indicated “extremely unlikely” and 5 “extremely likely.” Of the 8 patients who were unlikely or extremely unlikely to stop screening, 4 said that a recommendation to stop screening would not change their trust in the clinician while the other 4 said that it would make them trust the clinician less.

Patients were seeing a relevant specialist (ie, gynecologist for breast cancer screening, gastroenterologist for colorectal cancer screening, or urologist for prostate cancer screening) for 16 of the screening decisions. Among these, most (13 of 16 [81%]) considered the specialists’ recommendation about cancer screening as more important than their primary care clinicians’ if the recommendations were different.

## Discussion

This study adds to the existing literature on clinician decision-making about cancer screening in older adults using the method of medical record–stimulated recall, simultaneously eliciting perspectives from clinicians and patients, and focusing on screening cessation in older adults with limited life expectancy. Previous interview and survey studies examined factors important to clinicians in cancer screening decisions,^22,23,24,25,26,27,28,30,31,32^ which by study design asked the participants to deliberately reflect on the decision-making process. This study examined actual decisions made with specific patients, and found that screening decisions were often not the results of conscious deliberation. When the decisions were deliberate, they were affected not only by evidence-based factors such as patient age, health status, and family history but also by subjective factors such as the clinicians’ anecdotal experiences and the patients’ family and friends.

The findings of themes 1 and 2 are consistent with the dual-process theory of decision-making, which describes a slower logical way of processing information in contrast with one that is more automatic and unconscious.^42^ Our finding that the latter is involved in screening decisions in older adults is notable because clinical practice guidelines often recommend individualized decision-making regarding cancer screening in older adults,^9,10,11,12,13,14,15^ which implies deliberation, and many interventions to improve screening (eg, decision aids) assume deliberate decision-making.

In theme 2, we found that clinicians’ reliance on EMR alerts was associated with less deliberate decisions. An EMR-based intervention has been reported to be associated with reductions in prostate cancer overscreening.^43^ At the same time, potential bias from overreliance of EMR has been studied.^44^ Our result highlights both the potential opportunity to leverage the EMR to improve cancer screening practices as well as drawback if EMR alerts are not personalized to specific patients and clinicians become overly reliant on the alerts.

Although guidelines’ recommendation against routine cancer screening in older adults with limited life expectancy does not distinguish between different screening modalities,^9,10,11,12,13,14,15^ we found in theme 3 that clinicians often switched to less invasive screening options or using physical exam for screening, rather than stopping screening altogether, even when clinicians considered the standard screening tests (mammogram, prostate-specific antigen, colonoscopy) to be no longer appropriate. Although less invasive screening tests or physical exam pose less direct risk to patients, they can nonetheless be associated with unnecessary further tests and treatment that are more invasive and result in net harm. As cancer screening technology continues to evolve, with blood tests that can potentially detect multiple types of cancers,^45^ it is important to examine both the immediate and the long-term consequences from the screening test in the context of a patient’s life expectancy and competing risks of death.

In theme 4, the anecdotal experiences that the clinicians described are consistent with known cognitive biases.^46^ For example, a clinician who favors screening older patients after having had one 100-year old patient who did well with cancer treatment represents the availability bias.^46^ One prior study reported that clinicians were more likely to recommend breast cancer screening if they had encountered late stage cancer diagnoses in the absence of screening among prior patients, family or friends.^25^ Our results add to this limited evidence base by describing in more depth the type of anecdotes that clinicians found important and included both anecdotes that supported continuing screening and those that reinforced stopping screening. The utility of these anecdotal experiences in improving screening practices warrants further study.

Studies have reported that patients are enthusiastic about screening and that clinician recommendations are important in patients’ cancer screening decisions.^19,20,21,47^ It is unclear which of these 2 factors plays a more prominent role (ie, whether patients would be willing to stop screening if recommended by clinician). One study found that older adults were amenable to stopping screening in the context of a trusting relationship.^48^ Another study showed that among older patients who preferred to pursue colorectal cancer screening but were unlikely to benefit, only about one-third changed their minds after discussing screening with their clinicians.^49^ Focusing on older patients with recent screening, we found heterogeneity in their willingness to stop screening and that some patients who were reluctant to stop screening may react negatively to recommendation about screening cessation.

Our results may help inform future interventions aimed at reducing overscreening in older adults with limited life expectancy. First, effective interventions must overcome the inertia and promote more deliberate decision-making (eg, decision support tool outlining the benefits and harms of screening). Alternatively, the interventions based on behavioral economics that use nudges and defaults to promote desired behaviors may have utility.^50^ For example, EMR may incorporate patient life expectancy to suppress inappropriate screening alerts. Second, interventions may need to specifically address potential cognitive biases among clinicians, for example, giving more objective data to counter the effect of availability bias. Third, clinicians may benefit from information on patients’ willingness to stop screening ahead of a visit so as to tailor the discussion and target those who are more amenable. Fourth, interventions must recognize that the screening decision is not binary and explicitly address options of postponing the decision or using less invasive screening modalities. In addition, interventions that target not only the primary care clinician-patient dyad but also address specialists and social network (eg, family and friends) are likely to be more effective. Eliciting feedback from key stakeholders on how to feasibly incorporate these elements in interventions is a key next step.

### Limitations

This study has several limitations. First, we included a relatively small number of participants affiliated with 1 health system and their views may not be representative of clinicians and older adults in which practice settings, EMR usage, and sociodemographic characteristics are different. We are not able to comment on how clinician responses may have differed by specialty or other characteristics. However, qualitative studies are designed to gain rich in-depth information for hypothesis generation rather than generalizable data for large populations. Second, the cancer screening decisions used in medical record–stimulated recall, especially in those without recent screening, occurred months to years in the past which, if coupled with imperfect documentation, may have limited recall. Third, although medical record–stimulated recall minimizes recall bias, the responses are still subject to social desirability biases, especially as the investigator who conducted the interviews was acquainted with some of the participants. In addition, to get reciprocal perspectives from both patients and clinicians, we focused medical record–stimulated recall discussions on those patients who also responded to the telephone survey whereas cancer screening decision-making may have been different in non-responder patients.

## Conclusions

Results of this study found that cancer screening decisions were not always conscious or deliberate and were subject to multiple complex factors. Interventions to use strategies that facilitate more deliberate decision-making may be important to optimize screening in older adults with limited life expectancy.

## Notes

Supplement. **eAppendix 1.** Clinician Interview Guide **eAppendix 2.** Patient Survey Questions **eTable.** Alternative Approaches to Stopping Cancer Screening That Were Described by Clinicians (Theme 3) **eFigure.** Patient Recruitment Flowchart
