# Supplementary material for: Assessment of Clinician Decision-making on Cancer Screening Cessation in Older Adults With Limited Life Expectancy
Source: JAMA Netw Open. 2020 Jun 8;3(6):e206772. doi: 10.1001/jamanetworkopen.2020.6772 (PMC7280953; doi:10.1001/jamanetworkopen.2020.6772)
Supplement: Supplement. — eAppendix 1. Clinician Interview Guide eAppendix 2. Patient Survey Questions eTable. Alternative Approaches to Stopping Cancer Screening That Were Described by Clinicians (Theme 3) eFigure. Patient Recruitment Flowchart [file jamanetwopen-3-e206772-s001.pdf]

Supplementary Online Content

Schoenborn NL, Massare J, Park R, Boyd CM, Choi Y, Pollack CE. Assessment of clinician decision-making on cancer screening cessation in older adults with limited life expectancy. *JAMA Netw Open*. 2020;3(6):e206772.
doi:10.1001/jamanetworkopen.2020.6772

eAppendix 1. Clinician Interview Guide
eAppendix 2. Patient Survey Questions
eTable. Alternative Approaches to Stopping Cancer Screening That Were Described by Clinicians (Theme 3)
eFigure. Patient Recruitment Flow Chart

This supplementary material has been provided by the authors to give readers additional information about their work.

© 2020 Schoenborn NL et al. *JAMA Network Open.*

eAppendix 1. Clinician interview guide

We want to know how primary care providers think about cancer screening in older adults with limited life expectancy. We are interested in breast, colorectal, or prostate cancer screenings.

Clinical guidelines are changing – some use age while other use life expectancy as metrics to guide cancer screening. Some provider may find these guideline changes to be confusing. It is also sometimes unclear how the guidelines should be implemented in clinical practice. This is the reason we want your input as a primary care provider on what considerations are most important when you make screening recommendations to older patients (65 or older) and how patients' life expectancies are considered in that context. There is no right or wrong answer.

I will be asking about your thought processes for 2-3 specific patients of yours [Information about specific patients are customized for each clinician].

1. Thinking about patient #1 who had a screening test for XX cancer on [date], tell me how you came to this decision in this patient? How would you describe the way that decision was made?

2. Thinking about patient #2 who has not had YY cancer screening for several years, tell me how you came to this decision in this patient? How would you describe the way that decision was made? Do you have a general approach you use to council patients to stop screening? Do you think not ordering a cancer screening test affected your relationship with the patient in any way?

3. Thinking about these patients and your other older patients in general, what factors make you decide to stop versus continue ordering a mammogram/colonoscopy/PSA test? Which factor is the most important? - How do you weigh those factors?

4. How does your thought process differ for different types of cancer screening? Do you sometimes continue one type of cancer screening while stopping another type in the same patient?

5. Have there been times where you recommended against screening and patient still ended up getting the screening test?

6. Do you find that specialists are often involved in cancer screening decisions of your patients?

7. Some of the guidelines are now using life expectancy of the patient as a metric to guide when to stop screening – often using < 10 years as a threshold. What are your thoughts on the reasons for continued screening in older adults with <10 year life expectancy?

© 2020 Schoenborn NL et al. *JAMA Network Open.*

eAppendix 2. Patient survey

We are interested to know your opinions about cancer screening. There is no right or wrong answer. Please do not feel like you have to answer in a certain way.

1a. It looks like you recently had/ have not had a screening (mammogram/colonoscopy/PSA). Is that correct?
___ Yes
___ No
___ Not sure

(If response to Q1a was "No" or "Not sure")
1b. Are you planning to get a screening (mammogram/colonoscopy/PSA) at some point?
___ Yes
___ No
___ Not sure

2. Do you recall making a decision about whether or not to get screened for (breast/colorectal/prostate) with [clinician name]?
___Yes
___No

3. On a scale of 1-5, how satisfied are you with the decision about whether or not to get screening? If 1 = extremely dissatisfied and 5 = extremely satisfied?

_____

4. How much do you agree with this statement: "All in all, you have complete trust in your doctor." 1=strongly disagree and 5 = strongly agree

_____

(If response to Q1a <u>or</u> Q1b was "Yes")
5. If [clinician name] recommended that you stop getting (breast/colorectal/prostate) cancer screening and explained his/her reasons, how likely are you to go along with this recommendation? If 1 = extremely unlikely and 5 = extremely likely?

_____

© 2020 Schoenborn NL et al. *JAMA Network Open.*

(If response to Q1a or Q1b was "Yes")
6. If [clinician name] recommended that you stop getting (breast/colorectal/prostate) cancer screening and explained his/her reasons, would that affect how much you trust [clinician name]?
___ it would make me trust him/her less
___ it would make me trust him/her more
___ no change

7. On a scale of 1-5, how important is [clinician name]'s's recommendation when you make decision about cancer screening? If 1 = the least important among all factors that I consider and 5 = most important

______

8. Do you see a (gynecologist if discussing mammogram, gastroenterologist if discussing colorectal cancer screening, urologist if discussing PSA)?
_____Yes
_____No

(If response to Q8 is "Yes")
9. Whose recommendation would be more important when you make decision about cancer screening if the specialist and [clinician name] had different recommendations?
____ the specialist's
____ [clinician name]'s

10. Which best describes your health in general?

- ☐ Excellent
- ☐ Very Good
- ☐ Good
- ☐ Fair
- ☐ Poor

11. What is the highest grade of education that you completed?

- ☐ I did not finish high school, the highest grade that I completed: _____th grade
- ☐ I completed high school or have a GED
- ☐ <4 years of college
- ☐ I completed 4 years of college or have a higher post-graduate degree

© 2020 Schoenborn NL et al. *JAMA Network Open.*

eTable. Alternative approaches to stopping cancer screening that were described by clinicians (Theme 3).

Alternative approach	Example quote
Decreasing screening frequency	*"I have talked to some of older women over 80 about maybe not getting an annual mammogram, maybe going to every other year."*
Physical exam	"*If I have a patient with dementia, I'm not gonna recommend…screening test, I still will do a breast exam."*<br>"*I wouldn't do a PSA over 70…I'll do rectal exams regularly to check the prostate."*
Less invasive screening test	"*I don't see any reason not to screen people for blood in the stool but a colonoscopy for some of my patients their risk may be too high if they are pretty unstable."*
Deferring the decision to a later time	"*Usually they are so sick you don't really get to the health maintenance issues at all and I usually prioritize all of their other many issues during the visit…without ever taking [screening] off the list…it's usually always just pushed to the next visit without actually formally addressing it."*

© 2020 Schoenborn NL et al. *JAMA Network Open.*

eFigure. Patient recruitment flow chart.

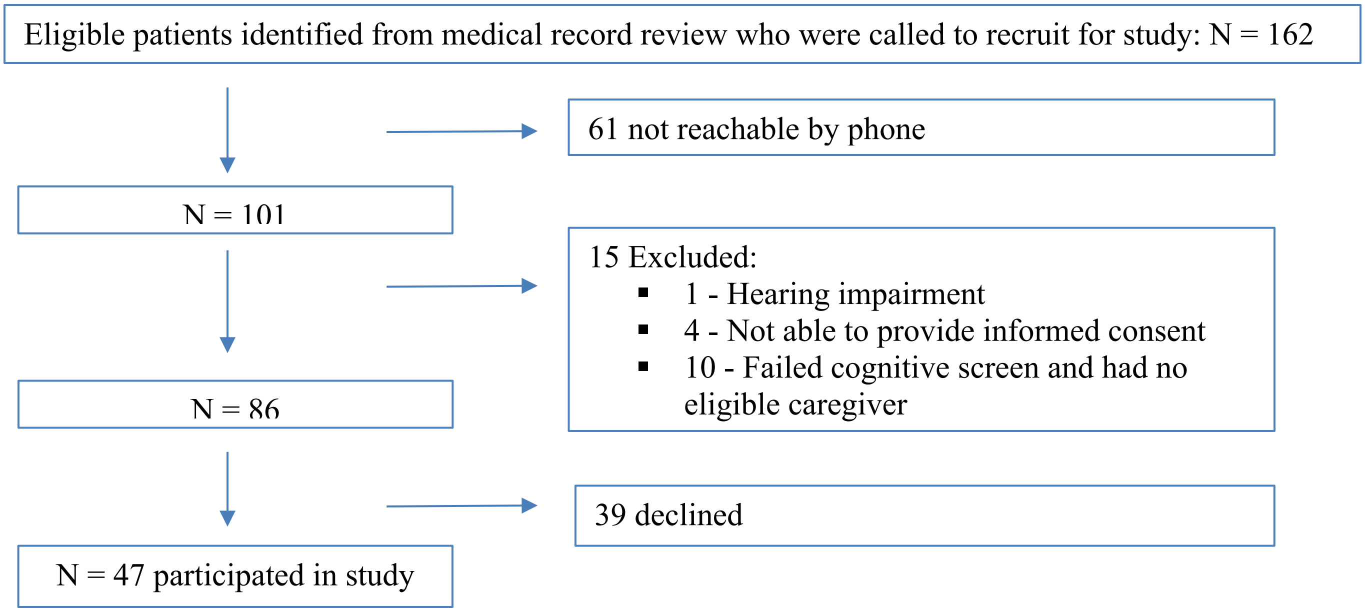

© 2020 Schoenborn NL et al. *JAMA Network Open.*
